# Impact of mindfulness training and yoga on injury and pain-related impairment: a group randomized trial in basic combat training

**DOI:** 10.3389/fpsyg.2023.1214039

**Published:** 2023-10-06

**Authors:** Carl D. Smith, Ian A. Gutierrez, Thomas H. Nassif, Kimberley L. Jordan, Kathryn M. Taylor, Amishi P. Jha, Amy B. Adler

**Affiliations:** ^1^Center of Military Psychiatry and Neuroscience, Walter Reed Army Institute of Research, Silver Spring, MD, United States; ^2^Center for Initial Military Training, Fort Eustis, VA, United States; ^3^Military Performance Division, U.S. Army Research Institute of Environmental Medicine, Natick, MA, United States; ^4^Department of Psychology, University of Miami, Coral Gables, FL, United States

**Keywords:** military, mindfulness, yoga, injuries, pain

## Abstract

**Introduction:**

Service members are at risk for pain-related difficulties in functioning and physical injury. Previous studies suggest that mindfulness training (MT) and yoga may prevent these outcomes. The present study was designed to determine the impact of MT and yoga on the health, pain, and injury of Army trainees completing 10 weeks of basic combat training (BCT).

**Methods:**

Platoons (≈40 trainees per platoon) were randomized to MT and yoga or training-as-usual in October to December 2020 at a large installation in the US. Self-reported outcomes were health, pain level, and pain impact on training, sleep, mood, and stress. Objective outcomes were injury-related medical encounters and number of diagnoses. The trial was registered at ClinicalTrials. Gov (NCT05550610).

**Results:**

Intervention trainees reported significantly better health (OR = 1.05, 95% CI [1.00, 1.10]) and less impact of pain on training (OR = 0.81, 95% CI [0.74, 0.90]), sleep (OR = 0.88, 95% CI [0.81, 0.95]), mood (OR = 0.86, 95% CI [0.78, 0.96]), and stress (OR = 0.88, 95% CI [0.79, 0.98]). There was no significant difference in injury-related medical encounters (AOR = 0.70, 95% CI [0.48, 1.03]), but intervention trainees had fewer diagnoses (OR = 0.67, 95% CI [0.47, 0.95]) and were 30% less likely to have a first medical encounter at any time during BCT. This difference emerged 3 weeks into BCT.

**Discussion:**

A combined MT and yoga intervention resulted in better trainee health. The US Army and other organizations requiring resilience under extreme stress should consider implementing MT and yoga to offset risks to employee health.

## 1. Introduction

The military places significant demands on service members to ensure their physical readiness for completing operational missions; however, sustained, intense physical activity can lead to injury and pain. In 2020, 49% of active-duty soldiers experienced a new injury, with most categorized as musculoskeletal injuries (MSKIs) (U.S. Army Public Health Center, 2022). Physical injuries are estimated to result in nearly 2 million medical encounters at treatment facilities and 10 million limited military duty days annually (U.S. Army Public Health Center, 2022). Furthermore, over 80% of service members with physical injuries have reported clinically significant levels of pain (Hauret et al., 2010; McGeary et al., 2016).

The substantial prevalence of injury and pain-related impairment in the military exerts a toll on service members and presents a profound risk to organizational readiness. Given that a history of physical injury increases risk for future injury (Sammito et al., 2021), preventing injury and effectively managing pain are critical to sustaining service member health. To this end, the U.S. Army has utilized a broad range of physical and behavioral protocols designed to monitor and reduce injuries, including implementing injury surveillance programs, improving training regimens, and testing the efficacy of orthotics (Jones et al., 2018; Molloy et al., 2020). In addition to injury prevention, programs designed to address the significant number of service members experiencing chronic pain from these injuries requires a resource-intensive interdisciplinary approach from medical, behavioral, and community providers (Vallerand et al., 2015). Despite some success with these interventions, injuries and injury-related pain remain common, underscoring the need for innovative and scalable approaches within the Department of Defense.

Mindfulness and yoga-based programs offer a novel means of mitigating the risk of injury and pain. Mindfulness, defined as “attending to relevant aspects of experience in a nonjudgmental manner” (Ludwig and Kabat-Zinn, 2008) may benefit musculoskeletal health and reduce pain through greater awareness of the body and attention to proper physical alignment. Although the direct relationship between mindfulness training (MT) and pain severity is not consistently observed (Hilton et al., 2017), research suggests that MT enables individuals to function even in the presence of pain (Reiner et al., 2013; Hilton et al., 2017). MT may also prevent injury by improving an individual’s focus on their physical movement and reducing distractibility (Birrer et al., 2012; Petterson and Olson, 2017). To date, research on MT in the military has predominantly examined attention, working memory, emotion regulation, and performance (Nassif et al., 2021; Hepner et al., 2022); however, little is known about the effects of MT on injury and pain among service members.

Yoga may also reduce the risk of injury and pain (Tran et al., 2001). Broadly, yoga includes a range of practices that often integrate physical postures, meditative practices, and spiritual concepts (Tran et al., 2001), For the present purposes, we use the term “yoga” to refer to modern postural yoga programs that focus on physical postures, breathing techniques, and mindfulness components (Tran et al., 2001). Yoga interventions can improve strength, endurance, and flexibility in healthy practicing participants (Tran et al., 2001; Raj et al., 2021). Randomized controlled trials have also found that yoga reduces pain in a variety of populations (Nambi et al., 2014; Kim, 2020), including veterans (Miller et al., 2017), but research has not examined the impact of yoga on injury and pain among service members. Additionally, most previous trials have relied on small cohorts (Kim, 2020).

Given their complementarity of approach, offering MT and yoga during the same interval may support holistic mind–body health by bolstering body awareness, reducing distractibility, enhancing flexibility, and attenuating the effects of pain. Such salutary effects may prevent injury and help service members maintain functioning. To investigate this possibility, we selected a venue for program implementation that ensured structured program delivery. Specifically, MT and yoga were delivered in tandem during basic combat training (BCT), a 10-week period of enculturation for newcomers to the military. We predicted that trainees receiving MT and yoga, compared to those receiving training-as-usual, would report better health, lower levels of pain, less frequent pain, less pain-related impact on training, sleep, mood, and stress, and would result in fewer medical encounters and injury diagnoses. This study is the first to examine the impact of MT and yoga on the physical health of service members in an intense military training environment. Identifying such benefits can directly inform training recommendations in the military and other physically demanding occupations where risk of injury is higher.

## 2. Materials and methods

### 2.1. Design

**Figure 1:**
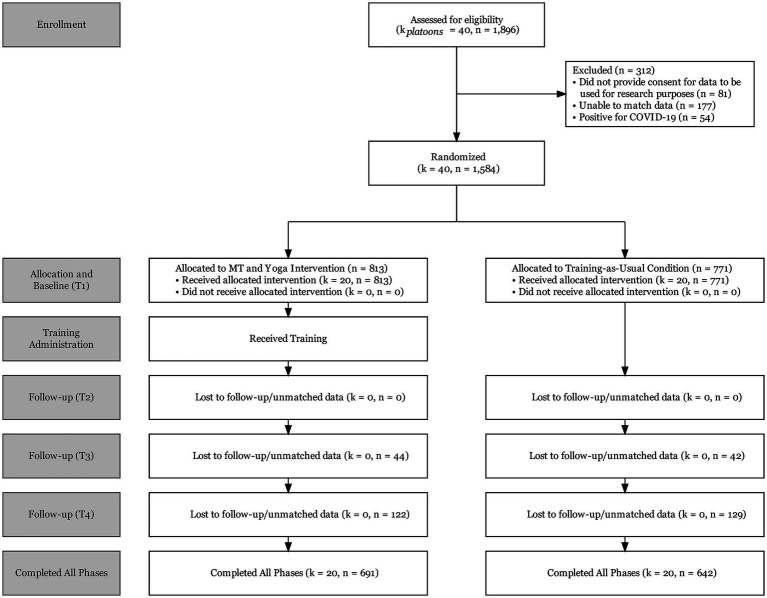
CONSORT diagram.

Trainees (*N* = 1,896) entering U.S. Army BCT between October and December 2020 were assigned to platoons (~40 persons), and platoons were randomized to two conditions. Twenty platoons received MT and yoga, and 20 platoons received training-as-usual. Initially, small groups (i.e., residential bays), rather than platoons, were used as an organizing element in BCT to minimize the risk of infecting larger numbers of trainees with COVID-19 during the pandemic. Randomization occurred after trainees were assigned to a residential bay when they first arrived at BCT. After 3 weeks, these small groups were reconfigured into larger platoons, keeping assignment to training condition intact. All participants were briefed prior to study enrollment. The CONSORT diagram is presented in Figure 1. Trainees attending BCT at one military installation in the southeastern United States were eligible for inclusion. Data were excluded from 312 (16.5%) of the 1,896 trainees who participated in the evaluation: 81 did not provide consent for their data to be used for research purposes, 177 could not be matched to condition or had inconsistent identification data, and 54 had COVID-19 and were relegated to a separate platoon, leaving a final sample of 1,584 for analysis.

Four surveys were administered over the 10-week BCT period. The first survey (T1) was administered in the first week of BCT before MT and yoga began; the second (T2), third (T3), and fourth (T4) surveys were administered during weeks 4, 6, and 9, respectively. Survey completion was voluntary; there was no financial compensation for study participation. The evaluation was approved by the Walter Reed Army Institute of Research Human Research Protection Branch, and registered at ClinicalTrials. Gov, Identifier: NCT05550610. Non-physical health outcomes assessed through this evaluation are reported elsewhere.

### 2.2. Intervention

The mindfulness training component was delivered via a 4-week, 8-h intervention developed for military personnel referred to as Mindfulness-Based Attention Training (MBAT) (Jha et al., 2020). Participants received weekly 2-h MBAT sessions during the first 4 weeks of BCT. There was a new topic each week: breath awareness and focused attention skills, body awareness, open monitoring, and interpersonal connection. The MBAT course material encouraged participants to engage in impromptu mindfulness practice throughout the day. Six days a week throughout BCT, participants also listened together as a platoon to a 15-min audio recording as part of an evening mindfulness practice. MBAT was delivered by 10 civilians with graduate degrees in the field of performance psychology. They were trained to deliver MBAT by completing 26 h of training over the course of 12 weeks (Jha et al., 2020). There were two MBAT instructors assigned to each platoon. When the intervention platoons were engaging in MBAT-related activities, trainees in the training-as-usual platoons were directed to study their BCT materials.

The yoga component of the intervention consisted of hatha yoga postures (Tran et al., 2001). The program was based on conventional hatha yoga postural sequences selected by a yoga expert. These postures were taught prior to and immediately after daily physical training every morning, 6 days a week, for a total of 3 h per week. This process was provided in place of the Army’s traditional preparatory and recovery drills and required the same amount of training time. In preparatory drills, yoga postures such as sun salutations, crescent lunge and eagle pose were selected to engage major muscle groups. In recovery drills, yoga postures such as gate pose, reverse plank, and bridge pose were selected to release tension in major muscle groups. Yoga training sessions were taught by one yoga instructor per platoon. Drill Sergeants who had received familiarization training circulated to offer postural corrections. In all, there were 21 yoga instructors, all of whom were registered yoga teachers with at least 200 h and 2 years of teaching practice. The yoga instructors completed 8 h of training specific to the study curriculum and had to demonstrate their competency with the material. When the intervention platoons were engaging in yoga, trainees in the training-as-usual platoons were engaging in traditional preparatory and recovery drills, which include calisthenics for warming up and stretching for cooling down.

### 2.3. Measures

#### 2.3.1. Overall health

Self-reported overall health was measured with a single item from the SF-8 Health Survey (Ware et al., 2001) “Overall, in the past 2 weeks, how would you rate your health?” using response options from poor (0) to excellent (5) (Jones et al., 2018). The SF-8 is a widely used and validated self-report measure for overall health.

#### 2.3.2. Pain level

Pain level was assessed with a single item assessed using 11 response options adapted from existing measures (Hjermstad et al., 2011): “In the past 2 weeks, what has been your level of physical pain?” using responses options from no pain (0) to severe pain (10) (Reiner et al., 2013). Studies demonstrate that 11-point numeric rating scales are reliable and valid methods for measuring pain in a variety of settings (Hjermstad et al., 2011).

#### 2.3.3. Pain frequency

Pain frequency was measured with a single item developed by Toblin et al. (2014). The item, “In the past 2 weeks, how often have you experienced physical pain?” was rated on a 5-point scale from not at all/a few days (1) to constantly (5) (Jones et al., 2018). This approach to measuring pain frequency is consistent with similar measures validated in previous research (dela Cruz et al., 2014).

#### 2.3.4. Pain-related impact

Trainees were asked the degree to which their pain has impacted training, sleep, mood and stress using four items from the validated Defense and Veterans Pain Rating Scale (DVPRS) (Buckenmaier et al., 2013). Trainees rated how much pain interfered with their training and sleep with response options from does not interfere (0) to completely interferes (10) (Reiner et al., 2013), how much pain affected their mood with response options from does not affect (0) to completely affects (10) (Reiner et al., 2013), and how much pain contributed to their stress with response options from does not contribute (0) and contributes a great deal (10) (Reiner et al., 2013).

#### 2.3.5. Injury-related medical encounters and diagnoses

Medical encounter and diagnostic data were obtained through the Army’s Soldier Performance, Health, and Readiness (SPHERE) data repository. The SPHERE contains Army medical encounter data and has been used extensively in previous research (Amoroso et al., 2000; Bulzacchelli et al., 2014). Medical encounters were documented when trainees sought help from medical providers at a military treatment facility or other facility where they used their military medical insurance. Encounters during the trainee’s time in BCT were defined using International Classification of Diseases (ICD)-10 diagnoses related to injury. SPHERE data included date of the encounter and ICD-10 diagnoses, which were used to categorize injuries by type and location (Roy et al., 2022).

### 2.4. Statistical analysis

We used linear mixed models to examine how condition, time, and time-by-condition interactions explained variance in outcomes while accounting for systematic variance attributable to platoon. Binomial generalized linear mixed models were conducted to estimate adjusted odds ratios (AORs) for condition-wise differences in injury, injury type, and injury region. Poisson generalized linear mixed models were used to examine condition-wise differences in the frequency of injury-related medical encounters and number of injury diagnoses. Multilevel models were estimated using restricted maximum likelihood with bound optimization by quadratic approximation, and nested time within trainee, and trainees within platoons. Finally, a Cox proportional-hazards model was used to examine time to first injury-related medical encounter by condition.

Across conditions and time points, missing data ranged from 7.0% (overall health), to 8.0% (pain level). Missing observations were handled using listwise deletion for each model. Analyses were conducted in R v.4.1.0 (R Core Team, 2021). Multilevel models were estimated using the “lme4” package (Bates et al., 2015).

## 3. Results

### 3.1. Participants

Participants reflected a typical cohort of BCT trainees: 1,189 (75.1%) were between the ages of 18 and 24, and 1,142 (72.2%) were male. No significant baseline differences were found between conditions by age group, χ^2^(3) = 0.32, *p* = 0.956, or by gender, χ^2^(1) = <0.01, *p* = >0.999.

### 3.2. Self-reported health and pain

Summary statistics for measures of self-reported overall health, pain level, pain frequency, the perceived relationship between pain and training, sleep, mood, and stress are presented in Table 1 by time and condition. Table 1 also reports regression coefficients, *p* values, and odds ratios for multilevel time-by-condition interactions for each outcome.

**Table 1:** Overall health, pain level, pain frequency, and pain interference by time and condition.

	Condition										
	MT and yoga				Training-as-usual						
	Time 1	Time 2	Time 3	Time 4	Time 1	Time 2	Time 3	Time 4	Multilevel time-by-condition interaction		
Variable	*M* (SD)	*M* (SD)	*M* (SD)	*M* (SD)	*M* (SD)	*M* (SD)	*M* (SD)	*M* (SD)	*b*	*p*	OR [95% CI]
Overall health	3.58 (1.04)	3.47 (1.03)	3.51 (1.00)	3.59 (1.07)	3.55 (1.01)	3.34 (1.00)	3.37 (1.00)	3.38 (1.08)	0.05	0.034	1.05 [1.00, 1.10]
Pain level	2.59 (2.36)	3.64 (2.24)	3.03 (2.30)	2.87 (2.38)	2.38 (2.17)	3.55 (2.20)	3.11 (2.31)	2.98 (2.57)	−0.12	0.078	0.88 [0.77, 1.01]
Pain frequency	1.77 (1.20)	2.39 (1.37)	2.11 (1.32)	2.01 (1.24)	1.69 (1.13)	2.38 (1.35)	2.19 (1.30)	2.03 (1.25)	−0.03	0.268	0.97 [0.92, 1.02]
Pain interfering with training	1.83 (2.45)	2.18 (2.44)	1.70 (2.30)	1.45 (2.13)	1.57 (2.25)	2.12 (2.38)	2.00 (2.38)	1.77 (2.33)	−0.21	<0.001	0.81 [0.74, 0.90]
Pain interfering with sleep	0.95 (1.95)	1.41 (2.28)	1.28 (2.16)	1.24 (2.07)	0.81 (1.79)	1.26 (2.15)	1.46 (2.24)	1.45 (2.29)	−0.13	0.002	0.88 [0.81, 0.95]
Pain affecting mood	1.66 (2.49)	2.09 (2.57)	1.81 (2.46)	1.61 (2.31)	1.41 (2.29)	2.04 (2.58)	1.93 (2.52)	1.80 (2.51)	−0.15	0.010	0.86 [0.78, 0.96]
Pain contributing to stress	2.14 (2.86)	2.35 (2.93)	1.96 (2.73)	1.69 (2.52)	1.87 (2.67)	2.30 (2.85)	2.09 (2.71)	1.78 (2.59)	−0.13	0.029	0.88 [0.79, 0.98]

Regression coefficients and odds ratios derived from three-level multilevel linear regression models predicting each outcome from time, condition, time-by-condition interaction, and random effects terms for uncorrelated intercepts and slopes with time nested within soldier, and soldier nested within platoon.

#### 3.2.1. Overall health

Across time and condition, trainees reported a mean score between 3 and 4, indicating “good” to “very good” health. Whereas mean overall health scores remained stable in the MT and yoga condition, changing by just 0.01 from Time 1 to Time 4, there was a mean decrease of 0.17 in the training-as-usual condition. The significant time-by-condition interaction for overall health scores indicated a difference in slopes by condition, such that self-reported overall health worsened in the training-as-usual condition relative to the MT and yoga condition.

#### 3.2.2. Pain level and frequency

Self-reported pain levels ranged between 2 and 4 among trainees, indicating a low to moderate level of pain. Pain increased by a mean of 0.28 in the MT and yoga condition and a mean of 0.60 in the training-as-usual condition. The time-by-condition interaction demonstrated that pain increased more in the training-as-usual condition compared to the MT and yoga condition; however, the condition-wise difference was not significant (see Table 1). Similarly, mean scores for self-reported frequency of pain hovered around 2, indicating that most trainees experienced pain several days per week across time and condition. Mean scores for pain frequency increased by 0.24 in the MT and yoga condition and by 0.34 in the training-as-usual condition from Time 1 to Time 4. The time-by-condition interaction indicated no significant difference in pain frequency over time by condition.

#### 3.2.3. Pain-related impact

Means for self-reported pain-related impact on training, sleep, mood, and stress ranged from around 1 to 2 across measures, time points, and conditions, indicating that pain had a low level of impact on each of these outcomes. Mean pain-related interference in training decreased by 0.38 in the MT and yoga condition from Time 1 to Time 4, but increased by 0.20 in the training-as-usual condition. Mean pain-related interference with sleep increased by 0.29 in the MT and yoga condition over the course of BCT, and by 0.64 in the training-as-usual condition. Mean pain-related effect on mood decreased by 0.05 in the MT and yoga condition from Time 1 to Time 4, but increased by 0.39 in the training-as-usual condition. Lastly, mean pain-related contribution to stress decreased by 0.45 in the MT and yoga condition from Time 1 to Time 4, but only by 0.09 in the training-as-usual condition. There was a significant time by condition interaction for each of the pain-related variables.

### 3.3. Injury-related outcomes

#### 3.3.1. Injury-related medical encounters

Frequencies of injury types and injury regions by condition are presented in Table 2. Across conditions, 38.6% of trainees (*n* = 612), or more than one in three trainees, had at least one injury-related medical encounter over the course of BCT. Trainees in MT and yoga were 18.4% less likely to have had at least one injury-related medical encounter compared to training-as-usual, χ^2^(1) = 9.99, *p* = 0.002. After accounting for clustering by platoon with binomial generalized multilevel modeling, MT and yoga trainees did not significantly differ in the number of injury-related medical encounters compared to training-as-usual; *b* = −0.35, SE = 20, *p* = 0.073, CI [95%] = 0.70 [0.48, 1.03]. Additionally, Poisson generalized multilevel modeling found that there was no significant difference in the frequency of injury-related medical encounters between conditions; *b* = −0.28, SE = 0.15, *p* = 0.055, CI [95%] = 0.75 [0.56, 1.01].

**Table 2:** Injury-related medical encounters, injury type, and injury region by condition.

		Condition		
	Total (*n* = 1,584)	MT and yoga (*n* = 813)	Training-as-usual (*n* = 771)	
Variable	*N* (percent)	*N* (percent)	*N* (percent)	AOR [95% CI]
One or more injury-related medical encounters^a^	612 (38.6%)	283 (34.8%)	329 (42.7%)	0.70 [0.48, 1.03]
**Number of injury-related medical encounters per trainee** ^b^				0.75 [0.56, 1.01]
0	972 (61.4%)	530 (65.2%)	442 (57.3%)	
1	361 (22.8%)	181 (22.3%)	180 (23.3%)	
2	157 (9.9%)	59 (7.3%)	98 (12.7%)	
3	61 (3.9%)	28 (3.4%)	33 (4.3%)	
4 or more	33 (2.1%)	15 (1.8%)	18 (2.3%)	
**Number of injury-related diagnoses per trainee** ^b^				0.67 [0.47, 0.95]
0	972 (61.4%)	530 (65.2%)	442 (57.3%)	
1	229 (14.5%)	131 (16.1%)	98 (12.7%)	
2	163 (10.3%)	71 (8.7%)	92 (11.9%)	
3	72 (4.5%)	27 (3.3%)	45 (5.8%)	
4	73 (4.6%)	22 (2.7%)	51 (6.6%)	
5 or more	75 (4.7%)	32 (3.9%)	43 (5.6%)	
**Injury type** ^a^				
Pain	1,167 (76.4%)	500 (77.2%)	667 (75.9%)	1.03 [0.73, 1.45]
Tendonitis/bursitis	41 (2.7%)	14 (2.2%)	27 (3.1%)	0.67 [0.31, 1.46]
Strain	41 (2.7%)	21 (3.2%)	20 (2.3%)	1.44 [0.76, 2.73]
Fracture	31 (2.0%)	13 (2.0%)	18 (2.0%)	1.18 [0.44, 3.16]
Sprain	26 (1.7%)	11 (1.7%)	15 (1.7%)	1.03 [0.44, 2.40]
Open wound	5 (0.3%)	1 (0.2%)	4 (0.5%)	0.34, [0.04, 3.03]
Dislocation	3 (0.2%)	1 (0.2%)	2 (0.2%)	1.10 [<0.01, 551.0]
Cold weather-related	3 (0.2%)	1 (0.2%)	2 (0.2%)	0.68 [0.06, 7.49]
Traumatic brain injury	2 (0.1%)	1 (0.2%)	1 (0.1%)	1.36 [0.08, 21.74]
Other/not specified	208 (13.6%)	85 (13.1%)	123 (14.0%)	1.00 [0.63, 1.57]
**Injury region** ^a^				
Lower extremity	1,268 (83.0%)	523 (80.7%)	745 (84.8%)	0.75 [0.49, 1.15]
Spine	99 (6.5%)	47 (7.3%)	52 (5.9%)	1.28 [0.75, 2.17]
Upper extremity	94 (6.2%)	44 (6.8%)	50 (5.7%)	1.14 [0.57, 2.30]
Torso	19 (1.2%)	11 (1.7%)	8 (0.9%)	1.92 [0.70, 5.32]
Head and neck	7 (0.5%)	2 (0.3%)	5 (0.6%)	0.52 [0.07, 4.08]
Unspecified	25 (1.6%)	12 (1.9%)	13 (1.5%)	1.26 [0.57, 2.77]
Other/not specified	15 (1.0%)	9 (1.4%)	6 (0.7%)	2.17 [0.51, 9.32]

Percentages reported as a total of valid responses. Higher AORs across outcomes reflect greater likelihood of medical encounter or injury in the training-as-usual condition.

a AORs for one or more medical encounters (dichotomized), injury type, and injury region derived from binomial generalized multilevel model predicting dichotomized outcomes with soldiers nested by platoon.

b AORs for number of injury-related medical encounters and number of injury diagnoses derived from Poisson generalized multilevel models predicting dichotomized outcomes with soldiers nested by platoon.

#### 3.3.2. Injury-related diagnoses

In terms of injury diagnoses, most injury diagnoses made across all trainees were pain diagnoses (*n* = 1,167, 76.4%) and located in the lower extremities (*n* = 1,268, 83.0%); injury type and injury region did not differ as a function of condition (Table 2). After accounting for clustering by platoon, Poisson generalized multilevel modeling demonstrated that trainees in MT and yoga had significantly fewer diagnoses compared to training-as-usual, *b* = −0.40, SE = 0.18, *p* = 0.026, CI [95%] = 0.67 [0.47, 0.95].

#### 3.3.3. Time to first medical encounter

**Figure 2:**
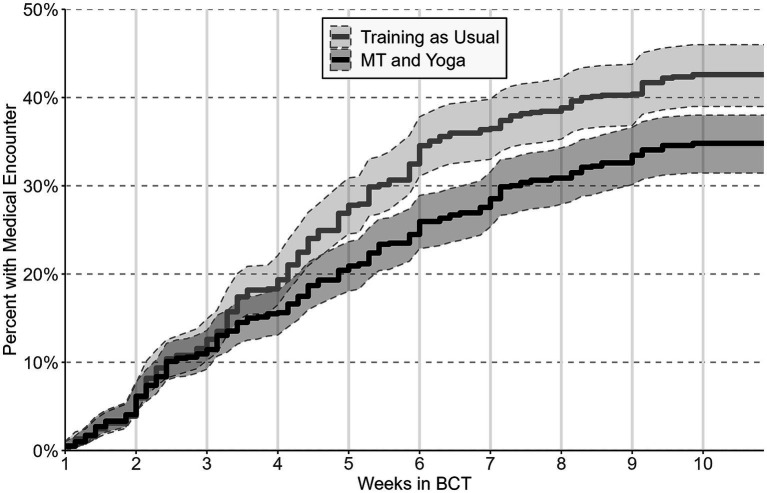
Inverted Kaplan-Meier survival curve depicting time to first medical encounter by condition with 95% confidence intervals.

The conditional Cox proportional-hazards model examined whether trainees differed in their likelihood of a medical encounter as a function of time. Findings from this model revealed a significant difference by condition, *b* = 0.26, SE = 0.08, *p* ≤ 0.001. The hazard ratio for condition was 1.30 (95% CI = 1.11, 1.52), indicating that trainees in the training-as-usual condition were 30% more likely than trainees in MT and yoga to have had at least one injury-related medical encounter at any time over the course of BCT. A visual assessment of the inverted Kaplan–Meier survival curve (Figure 2) shows that the proportion of trainees who had a first medical encounter began to diverge by condition between weeks 3 and 4 of BCT. Moreover, the proportion of trainees who had a medical encounter were significantly different by condition at the end of week 5, and remained so through the end of BCT.

## 4. Discussion

This study is the first to examine the effects of MT and yoga on the physical health of military service members. Results demonstrated that MT and yoga prevented a decline in self-reported overall health, attenuated the impact of pain on training, sleep, mood and stress, and reduced the likelihood of having an injury-related medical encounter for trainees at BCT, compared to training-as-usual. These findings align with previous reports that MT and yoga can benefit physical health (Tran et al., 2001; Birrer et al., 2012; Petterson and Olson, 2017; Raj et al., 2021).

In the case of pain, our findings demonstrated that while there were no significant differences in pain intensity levels over time by condition, military trainees appeared to benefit from MT and yoga in terms of how pain impacted them, a potentially relevant benefit given that the average pain rating was mild to moderate throughout BCT. These findings are consistent with previous studies that have suggested MT and yoga may reduce the degree to which pain interferes with functioning (Reiner et al., 2013; Nambi et al., 2014; Hilton et al., 2017; Kim, 2020). Given the emphasis on present-centered sensory awareness in mindfulness and yoga practices, the intervention may have helped trainees observe their discomfort without emotional reactivity, have greater awareness of their body in ways that helped them avoid injury, and use mindfulness practices and yoga postures that alleviated discomfort so that pain did not distract them from training, interfere with their sleep, disrupt their mood, or compound their stress.

Study results regarding medical encounters offer a nuanced pattern of findings. The most conservative statistical models demonstrated no overall significant difference between conditions with respect to the likelihood and frequency of injury-related medical encounters during BCT. However, trainees in the training-as-usual condition had significantly more injury diagnoses than trainees receiving MT and yoga, possibly suggesting the injuries may have been more complex. These results are consistent with previous studies demonstrating the impact of MT and yoga on injury prevention and physical well-being (Birrer et al., 2012; Petterson and Olson, 2017). Potential mechanisms of action underlying the observed injury results could include both physical and psychosomatic elements. For example, yoga is known to improve flexibility (Tran et al., 2001; Raj et al., 2021) and balance (Polsgrove et al., 2016), which may have prepared trainees in the intervention condition for the physical demands of BCT more than those receiving training-as-usual. Additionally, trainees receiving MT and yoga may have had fewer injury diagnoses because they were impacted less by pain than trainees in the training-as-usual condition, and thus, did not formally report as many medical concerns.

An analysis of the first injury-related medical encounter over time found that there was a 30% greater hazard of injury among MT and yoga relative to training-as-usual. Furthermore, the inverted Kaplan–Meier curves illustrate that the two conditions diverged at approximately 3 weeks, and this divergence was maintained through the remainder of BCT. The timing of this divergence is useful to consider. Previous research has indicated that physical health benefits require more than 2 weeks of MT (Cruze and Games, 2021) and several weeks of yoga practice (Park et al., 2020), suggesting that the benefits from the MT and yoga intervention also required weeks of practice.

Study strengths include a real-world occupational setting with subjective and objective outcomes. It is notable that results for subjective outcomes (self-reported health and pain-related impact) were corroborated by objective data that showed a meaningful condition-wise difference in injury-related medical encounters over the 10 weeks of BCT and a significant difference in the number of injury-related diagnoses between conditions. Study constraints include not being able to disentangle selective or additive effects of the two components of the intervention condition and not being able to determine mechanisms of effect. While this study did not examine causal mechanisms, future research may benefit from a multi-armed evaluation of intervention components and assessment of mitigating mechanisms. Such mechanisms could include the degree to which MT and yoga interventions contribute to strength, flexibility, body awareness, and emotion regulation (Tran et al., 2001; Busch et al., 2012; Veehof et al., 2016; Schmid et al., 2019; Raj et al., 2021).

Future research should also assess the effects of a MT and yoga intervention beyond BCT and identify ways to support the continuation of mindfulness and yoga practice once trainees have joined their first unit of assignment where group schedules may be less explicitly structured. Building on studies demonstrating the impact of practice on study outcomes, future research should consider optimizing practice engagement in an occupational setting (Blanck et al., 2018; Denkova et al., 2020, 2021; Nassif et al., 2021). Such studies can also examine the scope of effort required to sustain the benefits of MT and yoga in these settings. Given the potential resources that would be involved in a larger roll out of this kind of program, it would be important to evaluate methods for streamlining implementation, optimizing effects over time, and determining the cost–benefit of the investment.

## Data availability statement

The datasets presented in this article are not readily available because of institutional regulations related to human participant protection requirements but can be made available from the corresponding author upon reasonable request (may require data use agreements to be developed). Requests to access the datasets should be directed to CS, carl.d.smith179.mil@health.mil.

## Ethics statement

The studies involving humans were approved by Walter Reed Army Institute Human Subjects Protections Branch. The studies were conducted in accordance with the local legislation and institutional requirements. The participants provided their written informed consent to participate in this study.

## Author contributions

CS led the article writing, with substantial contributions and edits from TN, IG, AJ, and AA. IG conducted statistical analyses. KJ and KT provided support regarding data collection and analysis. All authors provided feedback on the final draft of the manuscript.
